# Riboflavin Attenuates Influenza Virus Through Cytokine-Mediated Effects on the Diversity of the Gut Microbiota in MAIT Cell Deficiency Mice

**DOI:** 10.3389/fmicb.2022.916580

**Published:** 2022-06-03

**Authors:** Ying Li, Chun-Wei Shi, Yu-Ting Zhang, Hai-Bin Huang, Yan-Long Jiang, Jian-Zhong Wang, Xin Cao, Nan Wang, Yan Zeng, Gui-Lian Yang, Wen-Tao Yang, Chun-Feng Wang

**Affiliations:** ^1^College of Veterinary Medicine, Jilin Agricultural University, Changchun, China; ^2^Jilin Provincial Key Laboratory of Animal Microecology and Healthy Breeding, Jilin Agricultural University, Changchun, China; ^3^Jilin Provincial Engineering Research Center of Animal Probiotics, Jilin Agricultural University, Changchun, China; ^4^Key Laboratory of Animal Production and Product Quality Safety of Ministry of Education, Jilin Agricultural University, Changchun, China

**Keywords:** MAIT cells, riboflavin, cytokines, influenza, gut microbiota

## Abstract

Influenza is a serious respiratory disease that continues to threaten global health. Mucosa-associated invariant T (MAIT) cells use T-cell receptors (TCRs) that recognize microbial riboflavin derived intermediates presented by the major histocompatibility complex (MHC) class I-like protein MR1. Riboflavin synthesis is broadly conserved, but the roles or mechanisms of riboflavin in MR1^–/–^ mouse influenza infection are not well understood. In our study, immunofluorescence techniques were applied to analyze the number and distribution of viruses in lung tissue. The amount of cytokine expression was assessed by flow cytometry (FCM), ELISA, and qPCR. The changes in the fecal flora of mice were evaluated based on amplicon sequencing of the 16S V3-V4 region. Our study showed that MAIT cell deficiency increased mortality and that riboflavin altered these effects in microbiota-depleted mice. The oral administration of riboflavin inhibited IL-1β, IL-17A, and IL-18 production but significantly increased the expression of IFN-γ, TNF-α, CCL2, CCL3, and CCL4 in a mouse model. The analysis of the mouse flora revealed that riboflavin treatment significantly increased the relative abundance of *Akkermansia* and *Lactobacillus* (*p* < 0.05) and decreased that of *Bacteroides*. In contrast, MR1^–/–^ mice exhibited a concentrated aggregation of *Bacteroides* (*p* < 0.01), which indicated that MAIT cell deficiency reduced the diversity of the bacterial population. Our results define the functions of MAIT cells and riboflavin in resistance to influenza virus and suggest a potential role for riboflavin in enhancing MAIT cell immunity and the intestinal flora diversity. Gut populations can be expanded to enhance host resistance to influenza, and the results indicate novel interactions among viruses, MAIT cells, and the gut microbiota.

## Introduction

Influenza A virus (IAV) is classified into various subtypes based on the protein structure and genetic characteristics of neuraminidase (NA) and hemagglutinin (HA) on the virus surface (Li et al., 2018). IAV is an acute infectious disease that causes sepsis in both humans and animals from the respiratory system to the whole body (Li et al., 2021). Examples of highly pathogenic influenza viruses are H7N9 (human) and H5N1(mice) (Yudhawati et al., 2020). H7N9 has caused epidemic outbreaks since 2016 in Guangdong, China (Bai et al., 2020). The direct lung damage caused by virus replication combined with a strong inflammatory response following infection determines the disease severity (Cardani et al., 2017). IAV recognition activates signaling pathways that recruit proinflammatory mediators and thereby activate T cells to recognize and kill the virus (Iwasaki and Pillai, 2014). The immune response is coordinated by a combination of proinflammatory cytokines and chemokines and results in effective clearance of the virus and restoration of homeostasis *in vivo* (Hemann et al., 2019).

Mucosal-associated invariant T (MAIT) cells are innate-like T cells that express TCRVα7.2-Jα33 in humans and Vα19-Jα33 in mice and identify microbial riboflavin derived intermediates presented by MR1 (Kjer-Nielsen et al., 2012). MAIT cells can be induced by the response to riboflavin synthesis, and the abundance of riboflavin can mark this subpopulation (Constantinides et al., 2019); these cells are enriched in tissues that come in contact with the external environment and symbiotic microbiota, including the liver (Nel et al., 2021), oral mucosa (Sobkowiak et al., 2019), respiratory system, intestine (Rouxel et al., 2017), and peripheral blood (PB) (Toubal et al., 2019). The assessment of the inflammatory pathological response of the disease may be due to bacterial-viral infection or stimulation by other proinflammatory factors. MAIT cells can rapidly metastasize to the site of inflammation when inflammation occurs, and MAIT cells are remarkably effective in defending against pulmonary Legionella infection (Ussher et al., 2014; Wang et al., 2018). Notably, peripheral-specific MAIT cells are activated in humans after infection with IAV, dengue virus, hepatitis C, and a series of acute infectious diseases, which drive the IL-18 dependent activation of MAIT cells and enhance the immune response (van Wilgenburg et al., 2016).

Vitamins not only have important functions in development and metabolism but also play a key role in the regulation of the immune system. The functions of vitamins D and A, two lipid-soluble vitamins, in regulating the immune response have been reported. Kjer-Nielsen et al. (2012) suggested a very different immune function for vitamins B2 (riboflavin) and B9 (folic acid), which are water-soluble vitamins. Riboflavin is considered an anti-inflammatory vitamin due to its antioxidant properties (Ahn and Lee, 2020). Bacteria that metabolize certain B vitamins produce molecules that activate MAIT cells. The authors propose that MAIT cells detect infected cells through vitamin metabolites and that vitamins can act as antigens that activate immune system T cells, and these hypotheses contribute to a more in-depth understanding of the immune system (Chua and Hansen, 2012). In conclusion, direct precursors and metabolites of riboflavin biosynthesis directly activate MAIT cells.

The gut microbiota is an intricate community of intestinal bacteria that plays critical roles in human health and disease development (Zhao et al., 2018). The maintenance of a symbiotic relationship with the gut flora is vital to human health. Disruption of this relationship can promote or even directly contribute to a multifarious variety of diseases and dysfunctions, including inflammatory diseases, colon cancer, and autoimmunity. Although various mechanisms through which the gut microbiota safeguards the host from intestinal infections have been described (McKenney and Pamer, 2015), the mechanisms through which the gut microbiota protects against extraintestinal infections, particularly respiratory infections, have not been fully elucidated (Brown et al., 2017).

Of note, we illustrated the tight relationship among the four components of MAIT cells, riboflavin, the abundance of the intestinal flora composition, and influenza virus infection. Our study provides the first demonstration that the infusion of riboflavin can influence the occurrence of influenza and that riboflavin changes the production of proinflammatory cytokines and chemokines. The riboflavin metabolic pathway of intestinal flora is involved in the regulation of influenza by MAIT cells through TCR-dependent signaling. An initial exploration of the potential mechanisms through which MAIT cells influence influenza development was performed.

## Materials and Methods

### Ethics Statement

All of the experimental procedures with mice were performed at the Experimental Animal Center under protocol number JLAU20210423001. The experiments were conducted under the supervision of the Experimental Animal Welfare and Ethics Committee of Jilin Agricultural University.

### Virus Strains and Mice

The strain used in the study was influenza A/Puerto Rico/8/1934 (H1N1). The mice were housed in a specific-pathogen-free (SPF) facility at the Laboratory Animal Center of Jilin Agriculture University (Changchun, China) with a 12-h day/12-h night light cycle, 40% humidity, and a temperature of 22–24°C and provided sterile water and feed. Six to eight-week-old female wild-type (WT, C57BL/6J) mice were obtained from Beijing Vital River Laboratory Animal Technology Co., Ltd., China. MR1-knockout (KO, MR1^–/–^) mice on the C57BL/6 background were provided by Shanghai Model Organisms Center, Inc. The mouse genotypes were determined by tail DNA PCR at the MR1 locus using the following primers: Fwd (wild type), TAATAAAATAAATCTTGGGACTGG; Fwd (knockout), CCCATATACGCTACTTCTA, Rev, TAATAAA ATAAATCTTGGGACTGG. The mice were anesthetized with 1% pelltobarbitalum natricum and intranasally administered 30 μl of influenza A/Puerto Rico/8/1934 H1N1 virus or sterile phosphate buffered saline (PBS, mock group).

### Experimental Design

The mice were randomly arranged into four groups: WT PBS group, riboflavin-pretreated WT group, MR1^–/–^ PBS group, and riboflavin-pretreated MR1^–/–^ group. The four groups of mice were placed in different cages and fed the same food and water. At 6 weeks of age, the mice were orally administered riboflavin (Solarbio, China, 100 mg/kg) or 30 μl of PBS every day for 7 days. All the mice were euthanized 7 days after the administration of 100 CFUs of H1N1 in saline or saline. Blood and feces were collected. Lymphocytes were isolated from lung tissue sites for flow cytometry, and feces were assessed by 16S sequence analysis. The lungs were prepared and stored in 4% paraformaldehyde. To explore the role of the gut microbiota in male C57BL/6J mice during PR8 infection, we depleted the gut microbiota using broad-spectrum antibiotics (Abx). Eight-week-old male C57BL/6J mice were treated with 10 mg each of ampicillin, neomycin, metronidazole, and vancomycin (167 mg/ml) daily for 5 days via oral gavage. Subsequently, the mice received Abx (1 g/l ampicillin, 1 g/l metronidazole, 1 g/l neomycin sulfate, and 0.5 g/l vancomycin, BioChemPartner, China) in their drinking water and riboflavin or PBS for 5 days. The antibiotic-treated water was then stopped 2 days, and the mice were then infected with IAV. The animal management procedures and all laboratory procedures abided by the regulations of the Animal Care and Ethics Committees of Jilin Agriculture University, China.

### Histopathology

The lung tissues of mice were fixed in 4% paraformaldehyde and dehydrated gradually in ethanol, and 3-μm-thick sections of the tissues were then stained with hematoxylin and eosin (H&E).

### Immunofluorescence Assay and Viral Signal Quantification

Paraffin sections were subjected to three 5-min washes with xylene and two washes with anhydrous ethanol and then fixed with 90% ethanol, 70% ethanol, and distilled water. The slides were blocked for 1 h in blocking buffer (3% normal goat serum, 1% BSA, and 0.3% Triton X-100 in sterile PBS). After blocking, recombinant influenza A H1N1 HA/hemagglutinin antibody rabbit mAb (Sino Biological, Beijing, diluted 400 times) was added to the blocking buffer, and the slides were incubated overnight at 4°C and washed three times with sterile PBS. The slides were then incubated with donkey anti-rabbit IgG labeled with FITC (BioLegend, United States) as a secondary antibody at 1:400 dilution for 1 h at room temperature, washed three times with immunostaining washing solution (Beyotime, Shanghai), and stained with 4,6-diamidino-2-phenylindole (DAPI) for 5 min at room temperature. The slides were washed three times with immunostaining washing solution. Eventually, antifade mounting medium (Beyotime, Shanghai) was used for sealing, and the sections were imaged with a Leica DM4B forward fluorescence microscope (Leica, Germany). The results were analyzed using ImageJ (Bitplane).

### Intracellular Staining and Flow Cytometry

The lungs were cut up and incubated in 1 ml of RPMI 1640 containing 1 mg/ml collagenase IV (Sigma, United States) for 45 min in a water bath at 37°C. The cells were passed through a 70-μm cell strainer and washed with RPMI with 10% FBS. Tissue was added to ACK lysis buffer (Beyotime, Shanghai) for 5 min and centrifuged. To assess the cytokine immune response, the lungs were isolated from each group of sacrificed mice. Individual cells from the lungs were collected and incubated in 24-well plates (2 × 10^6^ cells/ml per well). Purified PMA and NP proteins were added, and the cells were incubated in a CO_2_ incubator at 37°C for 12 h. Brefeldin A was added for the final 4 h of incubation. Cell supernatants were collected (100 μl) to determine the levels of cytokines. The cells were preincubated with anti-CD16/CD32 mAb on ice for 5 min and then with a LIVE/DEAD Fixable Near-IR Dead Cell Stain Kit (APC-Cy7) for 10 min. The individual cells were then incubated with CD3 (PerCP-Cy5.5), CD4 (FITC), and CD8 (APC) for 25 min at 4°C. Monoclonal antibodies were purchased from BioLegend. The cells were fixed, permeabilized with Cytofix/Cytoperm buffer (BD Bioscience, United States), immobilized, and stained with IFN-γ (PE-Cy7) and TNF-α (PE) antibodies. Ultimately, the cell samples were analyzed using an LSRFortessa analyzer (BD Biosciences, United States), and the data were analyzed using FlowJo software (Tree Star).

### Enzyme-Linked Immunosorbent Assay

The levels of secreted cytokines in serum, cell supernatant, and bronchoalveolar lavage fluid (BALF) were determined. Cell supernatant was obtained from individual cells isolated from the lungs of mice treated with purified PMA and NP proteins and incubated for 12 h, and the cell culture medium supernatant was aspirated. The mice were euthanized, and 1 ml of PBS (sterile) was then injected into the lungs and collected three times to obtain BALF. A quantitative analysis was conducted using commercial MeiMian ELISA kits (MeiMian Industrial Co., Ltd, Yancheng, China) in accordance with the manufacturer’s instructions.

### Quantitative Real-Time PCR

Total RNA in the lung was quantified using a MiniBEST Universal RNA Extraction Kit according to the manufacturer’s instructions (Takara, Japan). The concentration of RNA was measured with a BioTek Epoch 2 microplate reader (BioTek, United States). Total RNA was reverse-transcribed to cDNA using a PrimeScript™ RT reagent Kit with gDNA Eraser (Takara, Japan) according to the manufacturer’s instructions. Real-time PCR was performed with *Premix Ex Taq™* (Probe qPCR) (Takara, Japan). The specific primer sequences used in the experiment are summarized in Supplementary Table 1. The expression of genes of interest relative to that of β-actin was calculated using the 2^–ΔΔ^ ^Ct^ method.

### 16S rRNA Gene Sequencing and Analysis

Amplification of the V3-V4 hypervariable region of the bacterial 16S rRNA gene was performed using the 341F (5′-CCTAYGGGRBGCASCAG-3′) and 806R (806R: 5′-GGACTACNNGGGTATCTAAT-3′) primers. For Illumina NovaSeq analysis, a small fragment library was constructed, and the library was sequenced by double-end sequencing (Paired_End) based on the Illumina NovaSeq sequencing platform. After read splicing and filtering, operational taxonomic unit (OTU) clustering, species annotation, and abundance analysis, and deep data mining based on alpha diversity and beta diversity analyses were performed.

### Statistical Analysis

For the assessment of statistical significance, one-way ANOVA tests were performed using GraphPad Prism (version 9.0, United States), and the data are expressed as the means ± SEMs. *p < 0.05, **p < 0.01, ***p < 0.001, and ****p < 0.0001.

## Results

### MR1 Deficiency Is Associated With Enhanced Mortality

**FIGURE 1:**
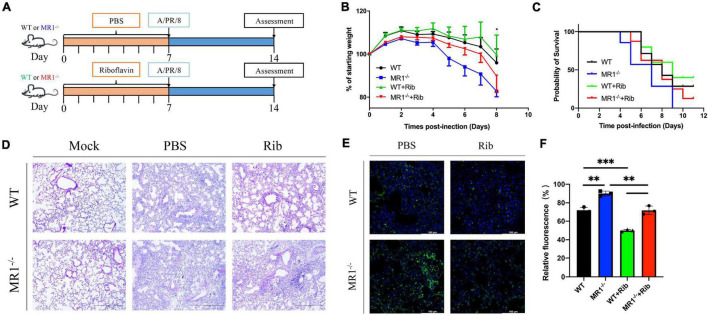
Mice show enhanced weight loss and mortality in response to influenza, and pretreatment with riboflavin improves their survival and reduces lung damage. **(A)** Schematic of the protocol: WT and MR1^–/–^ mice were administered riboflavin (100 mg/kg) or PBS by oral gavage for 1 week, and all the mice were then infected with 100 PFUs of H1N1. **(B)** Kinetic measurement of weight loss presented as a percentage and survival rate were analyzed by the log-rank test **(C)**. **(D)** Pulmonary histology during influenza virus infection (magnification, 200×). Scale bar, 400 μm. **(E)** Immunofluorescence analysis of lung influenza virus colonization load counts. H1N1 HA antibody (green) and nuclear (blue) expression were measured through lung staining and immunofluorescence techniques. The lung scale bars represent 100 μm **(F)**. The viral loads are depicted in the bar charts in the form of relative fluorescence intensities. The statistical significance was assessed by one-way ANOVA (*n* = 3 mice per group). *p < 0.05; **p < 0.01; and ***p < 0.001.

To examine the correlation between MAIT cells and riboflavin efficacy *in vivo*, we assessed the weight loss following IAV infection in WT and MR1^–/–^ mice and in WT and MR1^–/–^ mice that received oral riboflavin (Figure 1A). Following challenge with 100 PFUs of PR8 virus, the MR1^–/–^mice showed a higher body weight loss and increased mortality (Figures 1B,C) than the WT mice. Importantly, the weight loss in the MR1^–/–^ mice was improved by oral gavage with riboflavin 1 week prior to IAV infection. Consistent with this observation, severe tissue destruction and inflammatory infiltrates were observed in the lungs of untreated PR8-infected mice (Figure 1D). This lung tissue damage and inflammation were alleviated after riboflavin treatment, and the alleviation in WT mice was superior to that observed in MR1^–/–^ mice. The fluorescence-labeled virus was mainly retained in the lungs (Figure 1E), which indicated that the lungs constitute a main site of PR8 infection. Consistently, the strongest fluorescence intensity was observed in the lungs of MR1^–/–^ mice after the administration of Fluo-loaded PR8 HA (Figure 1F). Broadly speaking, these findings suggest that riboflavin can be orally administered in cases of milder infections.

### Effect of Mucosa-Associated Invariant T Cell Deficiency and Riboflavin on Cytokines and Chemokines in Response to Influenza Infection

**FIGURE 2:**
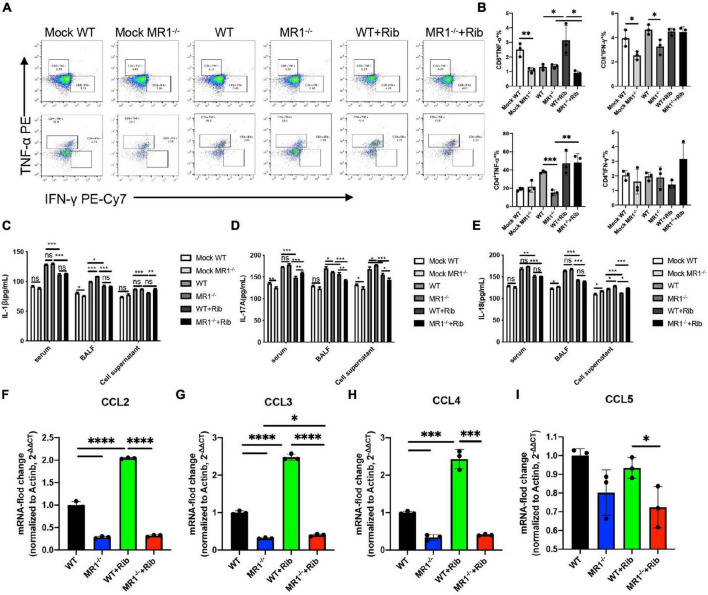
Riboflavin pretreatment reduces the PR8-induced proinflammatory status in WT and MR1^–/–^ mice. Mouse lungs were collected, and cell suspensions were prepared using a total of 2 × 10^6^ cells for incubation in plates containing purified PMA and NP proteins. The plates were incubated for a total of 12 h. Scatter plots of CD3^+^CD4^+^IFN-γ^+^TNF-α^+^ and CD3^+^CD8^+^IFN-γ^+^TNF-α^+^ **(A)** as percentages of IFN-γ and TNF-α cytokines in CD4^+^ and CD8^+^ T cells **(B)**. The serum, BALF, and cell supernatant of mice were collected, and ELISA kits were used for the analysis of IL-1β **(C)**, IL-17A **(D),** and IL-18 **(E)**. The chemokines CCL2 **(F)**, CCL3 **(G)**, CCL4 **(H),** and CCL5 **(I)** in the lungs were detected by qPCR. The data are presented as the mean values ± SEMs. *p < 0.05, **p < 0.01, ***p < 0.001, and ****p < 0.0001.

The cytokine-mediated immune responses in mice infected with H1N1 influenza virus were assessed because cellular immune responses in mice are thought to play a key role in the immunosuppression of IAV challenge. The activation of T cells in the lungs was detected by flow cytometry *in vitro* (Supplementary Figure 1). Significantly higher levels of IFN-γ and TNF-α were observed in CD3^+^CD4^+^ and CD3^+^CD8^+^ T cells in lung tissue after the oral administration of riboflavin (Figure 2A). We discovered that this protection depends to a large extent on the contribution of CD3^+^CD4^+^ T cells (Figure 2B). We investigated whether riboflavin could block cytokine production in mice during PR8 infection. The levels of IL-1β, IL-17A, and IL-18 secreted in serum, BALF, and cell supernatant were measured by ELISA (Figures 2C–E). The results revealed the following: infection with PR8 induced the production of IL-1β, IL-17A, and IL-18; the BALF expression of IL-1β in WT mice infected with the virus was lower than that in infected MR1^–/–^ mice (*p* < 0.001); the BALF expression of IL-17A in WT mice infected with the virus was higher than that in infected MR1^–/–^ mice (*p* < 0.05); and the expression of IL-17A and IL-18 in the supernatant of cells from WT mice infected with the virus was lower than that in the supernatant of cells from infected MR1^–/–^ mice (*p* < 0.05). The production of these cytokines was significantly attenuated after gavage with riboflavin, and cytokine expression in WT mice infected with the virus was lower than that in infected MR1^–/–^ mice. The levels of CCL2, CCL3, and CCL4 were also significantly increased after gavage with riboflavin (Figures 2F–H). The level of CCL5 in riboflavin-pretreated MR1^–/–^ mice infected with the virus was lower than that in riboflavin-pretreated WT mice (Figure 2I). In fact, the expression of these cytokines was higher in WT mice pretreated with riboflavin, and riboflavin administration had at most a marginal effect in MR1^–/–^ mice.

### Influenza Virus Infection Changed the Composition of the Gut Microbiota in Mice

**FIGURE 3:**
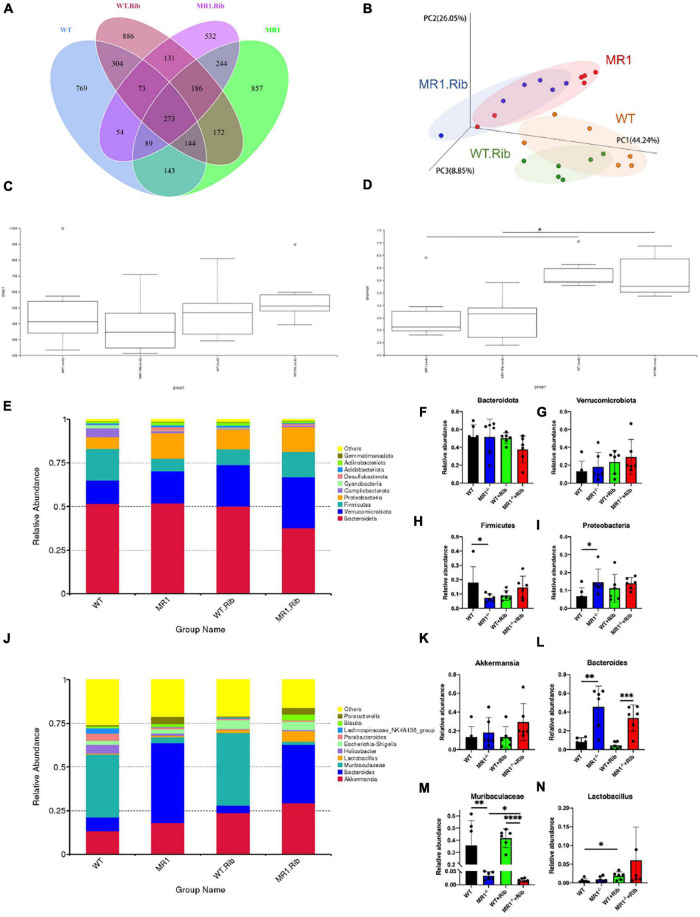
H1N1 infection alters the gut microbiota composition. **(A)** Venn diagram of the alpha diversity. Each circle represents a group of samples, and the number of overlapping circles represents the number of OTUs shared between groups. **(B)** PCoA based on weighted UniFrac distances. A closer sample distance indicates greater similarity in the species composition structure. Analysis of Chao1 diversity **(C)** in the feces of mice. **(D)** Shannon diversity in the feces of mice. **(E)** Relative abundance analysis of the top ten phyla in the fecal microbiota. Average bacterial taxonomic profiling at the phylum level. *Bacteroidota* **(F)**, *Verrucomicrobiota* **(G)**), *Firmicutes* **(H)**, and *Proteobacteria* **(I)** were detected in fecal flora through a LEfSe analysis of relative abundance. **(J)** Genus-level composition of the fecal microbiota. Relative abundance analysis of *Akkermansia* **(K)**, *Bacteroides* **(L)**, *Muribaculaceae* **(M),** and *Lactobacillus* **(N)**. *p < 0.05, **p < 0.01, ***p < 0.001, ****p < 0.0001.

We examined whether influenza infection could influence the gut microbiota. We analyzed the fecal pellets collected from the mice by 16S rRNA gene sequencing. The rarefaction curve directly reflects the rationality of the amount of sequencing data and indirectly reflects the species abundance in the four groups. As presented in Supplementary Figure 2, the grouping was sufficient to describe the microbial diversity. Through a comparison with the Silva138 database with respect to species annotation and the counting of different taxonomic levels, we identified 3,969 OTUs, and 3,953 (99.60%) of these OTUs could be annotated to the database. The number of unique OTUs in the WT, MR1^–/–^ and both groups were 638, 878, and 1,858, respectively. Additionally, the numbers of unique OTUs in the riboflavin-pretreated WT group, riboflavin-pretreated MR1^–/–^ group, and both groups were 1,069, 528, and 1,714, respectively. The results indicated that the composition of the microflora was highest in the feces of the riboflavin-pretreated WT mice. The number of OTUs in the four groups with mutual interactions was 1,232 (Figure 3A). The microbiota structural changes were then extracted from the most dominant elements and structures based on multidimensional data using a series of eigenvector and eigenvector sorting analyses, including weighted UniFrac distance-based principal coordinate analysis (PCoA), which accurately reflected the degree of variation between samples. H1N1 infection caused significant differences in the biological populations among the four groups (Figure 3B). The gut microbiota diversity was compared among the four groups using the Shannon index and the Chao1 index. Interestingly, a significant difference in diversity was found between the gut microbiota of the MR1^–/–^ mice and the WT mice and between the riboflavin-pretreated WT mice and the riboflavin-pretreated MR1^–/–^ mice, and the differences in the gut microbiota diversity between the groups were independent of riboflavin administration (Figures 3C,D). To assess the differences induced by riboflavin on the intestinal microorganisms in mice, we sequenced the intestinal flora at the bacterial phylum taxonomic level (Figure 3E). Similar abundances of the *Bacteroidota* and *Verrucomicrobiota* phyla were observed in the four groups (Figures 3F,G). One striking difference was that the MR1^–/–^ mice had a lower abundance of *Firmicutes* and a higher abundance of *Proteobacteria* than the WT group (*p* < 0.05) (Figures 3H,I). No significant increase in the relative abundance of *Akkermansia* was detected at the genus level (Figures 3J,K), and MR1 gene deficiency led to the appearance of concentrated clustering of *Bacteroides* (*p* < 0.01) (Figure 3L). Riboflavin administration also decreased the *Muribaculaceae* abundance in the MR1^–/–^ group and increased the *Lactobacillus* abundance in the WT group. MAIT cell deficiency decreased the abundance of *Muribaculaceae* independently in the WT and MR1^–/–^ mice (*p* < 0.01) (Figures 3M,N).

### LEfSe Analysis of Landmark Commensal Species

**FIGURE 4:**
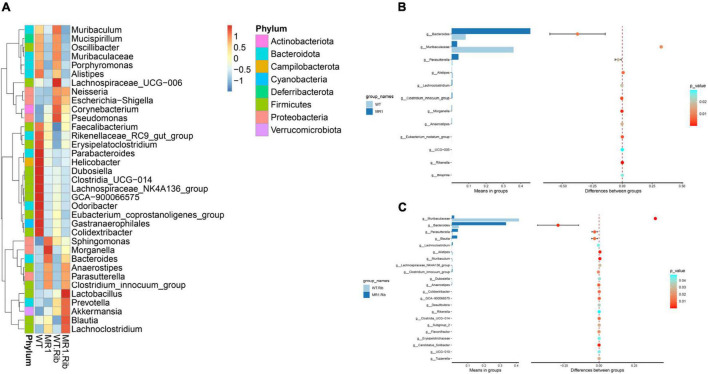
Examination of the differences in the gut microbiota between groups at the genus level. **(A)** Heatmap depicting the normalized abundance of each bacterial genus in individuals. **(B)** *T*-test of the species differences between the WT and MR1^–/–^ groups at the genus level. **(C)** *T*-test of the species differences between the riboflavin-pretreated WT and MR1^–/–^ groups at the genus level.

**FIGURE 5:**
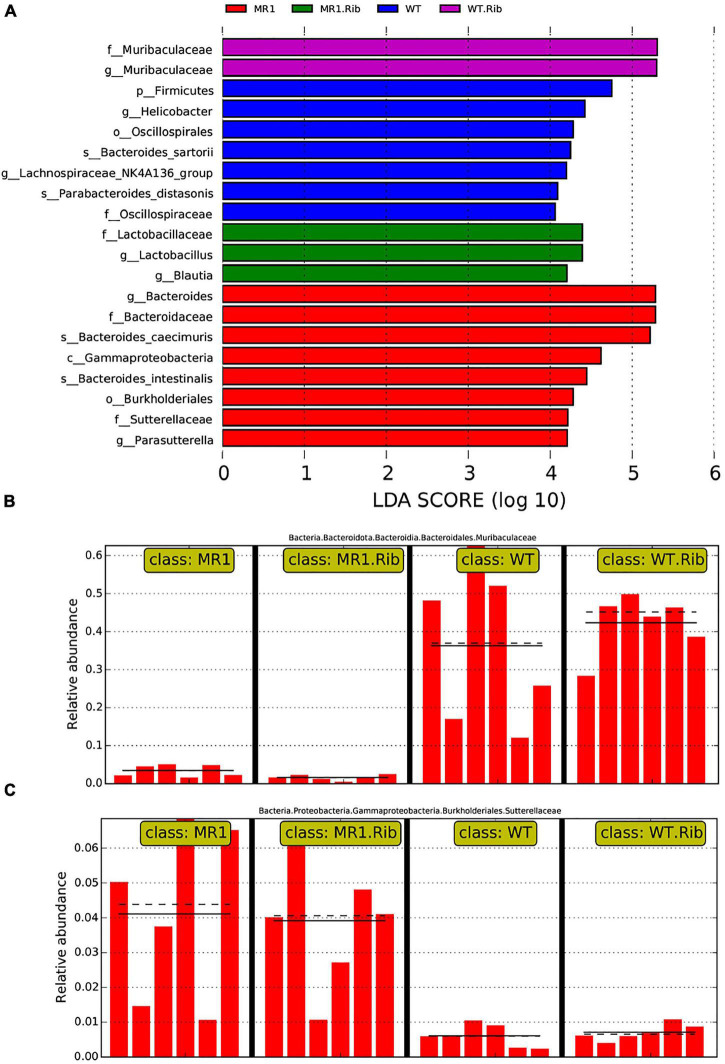
Biomarkers showing significant differences in species with latent Dirichlet allocation (LDA) scores greater than four between groups. The bar chart shows the distribution of the LDA values. **(A)** The bars represent species showing significant differences in abundance between different groups, and the length of the bars represents the magnitude of the effect of the species showing differences in abundance. Analysis of *Muribaculaceae* **(B)** and *Sutterellaceae* **(C)** in the different groups.

A heatmap was also constructed to display the microbial genera showing the largest differences in response to influenza infection in the four groups (Figure 4A). *Muribaculaceae* (phylum *Bacteroidetes*) was found at a significantly higher abundance in the riboflavin-pretreated WT mice and the WT mice (Figures 4B,C). The LEfSe branch diagram is shown in Supplementary Figure 3. *Blautia* (phylum *Firmicutes*) showed different trends among the four groups (Figure 5A). Homoplastically, the MR1^–/–^ group exhibited a greater abundance of *Bacteroidetes* than the riboflavin-pretreated MR1^–/–^ group. The LEfSe analysis showed that the abundance of *Muribaculaceae* (Figure 5B) was significantly decreased in the MR1^–/–^ and riboflavin-pretreated MR1^–/–^ groups compared with the WT and riboflavin-pretreated WT groups, whereas that of *Sutterellaceae* (Figure 5C) was significantly increased in MR1^–/–^ and riboflavin-pretreated MR1^–/–^ groups. Therefore, the absence of MAIT cells was identified as the factor driving the differences between the groups, and the results indicate that MAIT cells maintain intestinal homeostasis after H1N1 infection by interacting with commensal microorganisms.

### Correlation of Operational Taxonomic Unit Relative Abundances With Cytokines and Chemokines

**FIGURE 6:**
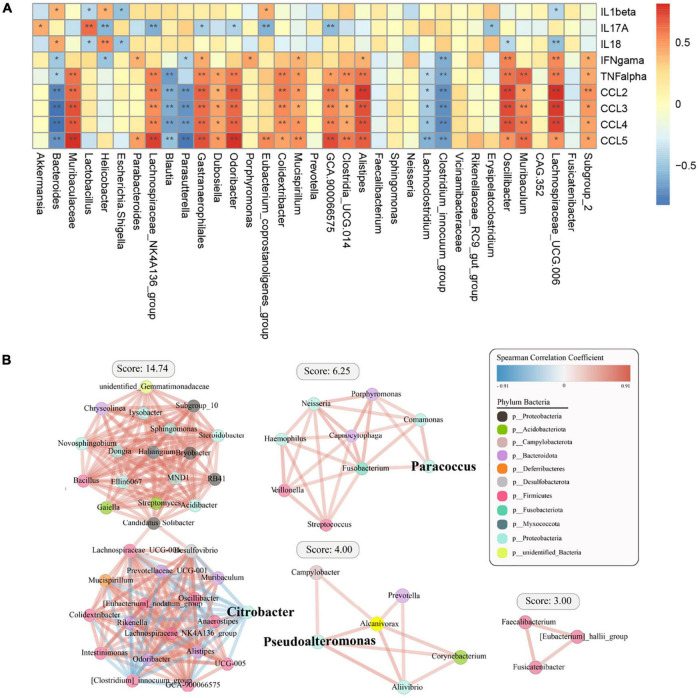
Spearman correlation analysis between specific or differential microbial taxa and infection-related indicators among the four groups. The shade of the resulting color is closely related to the value of the Spearman correlation coefficient (*p < 0.05, **p < 0.01, indicates a significant finding after correction for the false discovery rate). **(A)** The red and blue colors indicate positive and negative correlations in the relative abundance, respectively. **(B)** Correlation coefficient between the dominant bacterial phyla in the different groups.

We then analyzed the relationship between cytokines and chemokines and major OTUs among the four groups (Figure 6A). We researched 35 OTUs showing significant correlations between relative bacterial abundance and inflammatory mediators. Of note, IL-1β was positively correlated with *Bacteroides*, IL-17A was positively correlated with *Akkermansia* and *Lactobacillus*, IL-18 was positively correlated with *Helicobacter* and *Bacteroides*, and IFN-γ, TNF-α, and chemokines were positively correlated with *Muribaculum* and *Lachnospiraceae_UCG.006*. Conversely, IL-1β and IL-18 were negatively correlated with *Lachnospiraceae_UCG.006*, IL-17A was negatively correlated with *Helicobacter*, and IFN-γ, TNF-α, and chemokines were negatively correlated with *Bacteroides*. Overall, systemic immune responses were found to be associated with alterations in the intestinal flora. Interestingly, *Bacteroidetes* and *Lachnospiraceae_UCG.006* exhibited the strongest correlations, including both positive and negative correlations. In addition, a diagram of the correlations of OTUs with *Proteobacteria*, *Firmicutes*, and *Bacteroidota* was plotted to show the relationships among some important members belonging to the dominant phyla (Figure 6B).

### Metabolic Functional Kyoto Encyclopedia of Genes and Genomes Pathway Annotations Predicted With PICRUSt2

**FIGURE 7:**
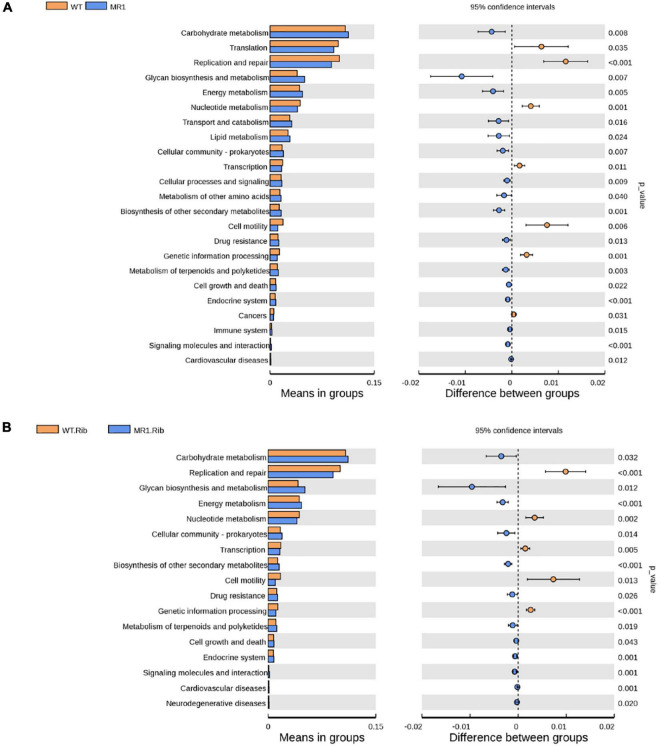
Differences in the abundances of KEGG pathways inferred by PICRUSt2. The abundances of the KEGG pathways encoded by the gut microbiota of the WT and MR1^–/–^ mice **(A)** and the riboflavin-pretreated WT and MR1^–/–^ mice **(B)**.

We observed significant differences in the predicted functional abundances between the gut microbiota using PICRUSt2. The identified KEGG pathways such as metabolism, genetic information processing, and organismal systems are shown in Supplementary Figure 4, and these include the metabolism of cofactors and vitamins, carbohydrate metabolism, and amino acid metabolism. Notably, many enriched pathways, such as lipid metabolism and carbohydrate metabolism, were less abundant in infected WT mice and riboflavin-pretreated infected WT mice than in their respective MR1^–/–^ counterparts. The infected WT mice showed a lower abundance of some essential pathways, such as immune systems, transcription, and replication/repair, than the infected MR1^–/–^ mice (Figure 7A). The riboflavin pretreatment of WT mice resulted in increased levels of pathways potentially related to metabolism, such as nucleotide metabolism, compared with the levels found in riboflavin-pretreated MR1^–/–^ mice (Figure 7B).

### Microbiota Depletion Decreases the Antiviral Capacity After Influenza Infection

**FIGURE 8:**
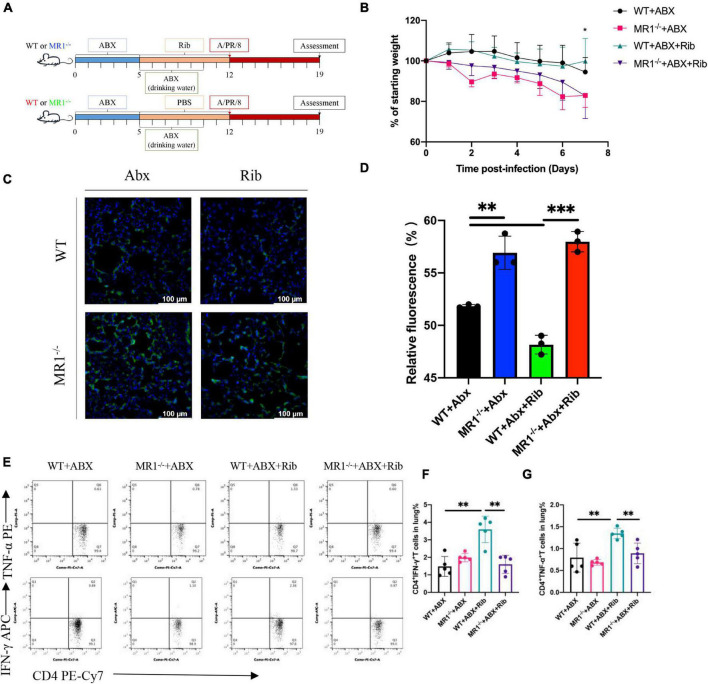
Depletion of the microbiota increase the IAV burden in tissues after infection. Flow diagram of the experiment. **(A)** The methods used for the antibiotic and riboflavin treatment of Abx-treated mice are described in the “Materials and Methods” section. The mice were then infected with IAV. The lungs were collected at 7 days, and the mice were weighed daily after infection. **(B)** Curve of the body weight of the WT mice and MR1^–/–^ mice. **(C)** Pulmonary histology during influenza virus infection (magnification, 200×). Immunofluorescence determination of influenza virus colonization load counts in the lungs. H1N1 HA antibody (green) and nuclear (blue) expression were measured by lung staining and immunofluorescence techniques. The lung scale bars represent 100 μm **(D)**. The viral loads are depicted in the bar charts in the form of relative fluorescence intensities. **(E–G)** Frequency of CD4^+^IFN-γ^+^TNF-α^+^ T cells in the lungs of the four groups at 7 dpi. Each experiment was repeated three times. The data are shown as the means ± SEMs. Statistical significance was determined using the Wilcoxon rank-sum test. *p < 0.05, **p < 0.01, ***p < 0.001.

To explore the role of the gut microbiota in mice during infection, we depleted the microbiota in mice using broad-spectrum antibiotics (Figure 8A). During the 1st week of antibiotic treatment, the mice lost weight, and at the end of the antibiotic treatment, significant weight loss was observed in the Abx-treated MR1^–/–^ mice compared with the WT mice (Figure 8B). The results showed that the IAV load in the lungs of the Abx-treated mice was increased, as shown in Supplementary Figure 5. However, riboflavin pretreatment partially reversed these changes (Figures 8C,D). Specifically, riboflavin significantly increased the expression of TNF-α and IFN-γ in Th1 well-differentiated CD4^+^ T cells of WT mice (*p* < 0.01), and the expression of TNF-α and IFN-γ in MR1^–/–^ mice was significantly lower than that in WT mice (*p* < 0.01) (Figures 8E–G). These data indicated that the microbiota helped enhance the antiviral capacity and that the absence of MAIT cells was the factor driving the differences between the groups.

## Discussion

Numerous studies have clarified that alterations in the intestinal flora play a role in the pathogenesis of multifarious infectious diseases. Recent studies have identified the functional properties of MAIT cells associated with enriched distribution in the lung, increased cytokine expression, and activation of riboflavin metabolites in the intestinal flora, and we hypothesized that MAIT cells regulate the intestinal flora and alter the composition of the microbiota during influenza development. Our study also highlights the importance of riboflavin in host defense against influenza virus infection. We demonstrated that MAIT cells can influence the development of influenza by regulating the composition of the intestinal flora microbiota and exerting immunosuppressive effects in mice after riboflavin gavage.

Microbial riboflavin synthesis and costimulatory signaling induce the activation and accumulation of MAIT cells after infection (Chen et al., 2017). MAIT cells have been used in clinical studies of both acute dengue virus and chronic hepatitis C virus (HCV) (Ussher et al., 2018). We found that HCV elicits a response to MAIT cell activation and is highly sensitive to T-cell-derived IFN-γ, which limits HCV replication *in vitro*. MAIT cells undergo functional and phenotypic changes during clinical and trial sepsis (Trivedi et al., 2020). During experimental sepsis, MAIT cells can be highly activated and contribute to cytokine responses that are protective against death. During COVID-19 infection, MAIT cells are also involved in the immune response against SARS-CoV-2 and in cellular immunopathogenesis (Parrot et al., 2020). These series of findings from viral infectious diseases could prove that MAIT cells play a protective role against respiratory and systemic viral diseases.

Cell-mediated immune responses are considered a crucial part of the challenge of immunity against IAV (Yang et al., 2017). After influenza virus infects the body, the pattern recognition receptor (PRR) encoded by the host germline gene recognizes the pathogen-associated molecular pattern (PAMP) of the pathogen, triggers antiviral immunity, activates multiple sets of cell signaling pathways, and induces cytokines (TNF-α, IFN-γ, IL-17A, and IL-1β) and chemokines (CCL2, CCL3, CCL4, and CCL5), which are released, thereby promoting the activation and chemotaxis of inflammatory cells, mediating inflammatory responses, and accelerating virus clearance, while persistent overexpression of inflammatory factors can lead to the activation of a large number of inflammatory cells, resulting in higher levels of inflammatory factors secretion, resulting in severe inflammatory response and tissue cell damage. MAIT cells activation could also be of importance for both protection and immunopathology (van Wilgenburg et al., 2016) in tissues such as the liver and lungs and exerts an antiviral effect, and the activation of these cells can occur in a direct or an indirect manner. Therefore, we hypothesize that the partial activation of MAIT cells in tissues could be directly related to antiviral function. Undoubtedly, the levels of proinflammatory mediators, such as TNF-α, NO, and IL-1β, in the plasma of septic mice are reduced after the intravenous administration of riboflavin (Toyosawa et al., 2004). Granzyme, IL-12, and perforin regulation play a vital role in the regulation of intracellular bacterial infections (Kurioka et al., 2015). Consequently, the induction of the CD3^+^CD4^+^ T immune response to IAV may reduce the course and infection of the clinical disease by maximizing the clearance of virus-infected cells in mice because the CD3^+^CD4^+^ T-cell response plays a basic role in the prevention of IAV infection. MAIT cells antecedently generate either type-1 (IFN-α-dependent early phase) or type-2 (IL-18-induced later phase)-associated cytokines (Koay et al., 2016; Flament et al., 2021). MAIT cells are also activated by microbe-derived antigens and produce cytokines, including IL-17A, and these effects are important in the maintenance of gut integrity (Lee et al., 2015). A transcriptome analysis of human and murine MAIT cells has revealed that a tissue repair profile, and tissue repair genes, including CCL3, are upregulated in human MAIT cells after their activation (Leng et al., 2019). The deficiency of MAIT cells shows marked upregulation of IL-1β, IL-17A, and IL-18, downregulation of CCL2, CCL3, and CCL4, and MAIT cells may play a considerable role in early antiviral protection in mice by secreting inflammatory cytokines and chemokines. Overall, the MR1-dependent and cytokine-mediated mechanisms underlying the anti-disease effects of MAIT cells will be essential for addressing issues related to clinical disease.

According to previous studies, H1N1 does not directly infect the gut (Wang et al., 2014), which raises the question of the interaction between respiratory infections and the gut flora. Based on the results reported by Bartley, lung tissue and gut mucosal surfaces are considered to share immunological signals (Bartley et al., 2017); hence, inflammation in one area may affect another area. In organisms, the gut is considered the largest immune area; therefore, signals from the gut microbiota play a crucial role in IAV infections. For instance, mouse enteric virus (Kuss et al., 2011) requires an effective infection mechanism involving intestinal bacterial flora. IAV infection temporarily transforms the fermentative activity and composition of the gut flora in mice (Sencio et al., 2020). Conversely, IAV will protect the body from triggering a more effective immune response in cases of an abundance of intestinal bacterial flora.

MAIT cells and riboflavin reduce infection by adjusting the components of the gut flora, and at the phylum level, we found that the predominant phyla included *Bacteroidota*, *Verrucomicrobiota*, and *Firmicutes*. Representative gut species tend to be strains of the phylum *Bacteroidota* (Hildebrand et al., 2021). At the genus level, reduced abundance of *Bacteroides* was observed. *Bacteroides* is an important symbiotic bacterium, and a decrease in its abundance may be responsible for riboflavin metabolites. Notably, in previous studies, a number of verified probiotics were found to exhibit a significant positive correlation with influenza infection (Yang et al., 2017). For instance, the abundance of *Akkermansia* was significantly increased at the later stages of infection in the riboflavin-pretreated WT and MR1^–/–^groups. Additionally, the beneficial role of *Akkermansiaceae* in the intestinal immune response may also play a complementary role in the fight against influenza infection (Hu et al., 2020). *Lactobacillus* are considered probiotics due to their health-promoting effects (Zhang et al., 2018). *Lactobacillus* has been found to exert a protective effect against airway inflammation during RSV infection (Fujimura et al., 2014).

The increasing amount of research on the gut microbiota against influenza performed in recent years has revealed that some strains exert a significant protective effect during the course of IAV infection (Zhang et al., 2020), and these strains include *Bifidobacterium longum* (Kawahara et al., 2015), *Lactobacillus rhamnosus* (Villena et al., 2012), and *Lactobacillus pentosus* (Kiso et al., 2013). Simultaneously, the decrease in their abundances observed ion the mice intragastrically administered riboflavin may be related to increases in certain species of the gut microbiota, such as *Lachnospiraceae* and *Muribaculaceae*. *Muribaculaceae* have diverse functions in the degradation of complex carbohydrates (Lagkouvardos et al., 2019). Different levels of *Lachnospiraceae* serve as the main predictor of the intestinal flora during the onset of H1N1 infections. In summary, further studies should explore the specific mechanisms underlying the changes induced by riboflavin. Riboflavin might be a novel treatment strategy for IAV. Each species of the microbiota sends signals to the immune system in very different manners (Geva-Zatorsky et al., 2017). In our study, the production of cytokines associated with influenza infection exhibited different correlations with each OTU. We noted that the more pronounced changes in the relative abundance of intestinal bacteria were related to the chemokines IL-17A and IL-1β, respectively. More broadly, the results demonstrate that the generation of cytokines relevant to H1N1 infection are differentially associated with each of the 35 OTUs identified in our study.

By combining the above-described results with previously published findings, we confirmed the relationship among riboflavin, intestinal flora, MAIT cells, and influenza. Stimulation of the fecal flora by cytokines and MAIT cell deficiency exacerbates viral colonization and aggravates infection. Riboflavin and MAIT cells can regulate the intestinal flora through the immune action of cytokines. Additionally, MAIT cells suppress the development of influenza by exerting immunosuppressive effects. Our research may provide a new clinical prevention and treatment strategy for influenza that comprises MAIT cell therapy, riboflavin therapy, and cytokine treatment and can yield improved disease treatment outcomes.

## Data Availability Statement

The raw reads were deposited into the NCBI Sequence Read Archive database under the accession number SRP359874 and the BioProject accession number PRJNA807116.

## Ethics Statement

All of the experimental procedures on mice were performed at the Experimental Animal Center under protocol number JLAU20210423001. Experiments were conducted under the supervision of the Experimental Animal Welfare and Ethics Committee of Jilin Agricultural University.

## Author Contributions

G-LY, W-TY, and C-FW contributed to the conception of the study. YL, C-WS, and Y-TZ contributed significantly to the analyses conducted in the study and the preparation of the manuscript. H-BH, Y-LJ, and J-ZW performed the data analyses and wrote the manuscript. XC, NW, and YZ helped to perform the analyses with constructive discussions. All authors contributed to the article and approved the submitted version.

## Conflict of Interest

The authors declare that the research was conducted in the absence of any commercial or financial relationships that could be construed as a potential conflict of interest.

## Publisher’s Note

All claims expressed in this article are solely those of the authors and do not necessarily represent those of their affiliated organizations, or those of the publisher, the editors and the reviewers. Any product that may be evaluated in this article, or claim that may be made by its manufacturer, is not guaranteed or endorsed by the publisher.
