# Supplementary material for: Riboflavin Attenuates Influenza Virus Through Cytokine-Mediated Effects on the Diversity of the Gut Microbiota in MAIT Cell Deficiency Mice
Source: Front Microbiol. 2022 Jun 3;13:916580. doi: 10.3389/fmicb.2022.916580 (PMC9204145; doi:10.3389/fmicb.2022.916580)
Supplement: Supplementary file 1 [file Table_1.DOCX]

Supplementary Material

| Supplementary Table 1. Primer sets used for quantitative reverse  transcription-PCR | |
| --- | --- |
| Gene | Primer |
| *CCL2* | Forward: AGCAGCAGGTGTCCCAAAGA |
|  | Reverse: GTGCTGAAGACCTTAGGGCAGA |
| *CCL3* | Forward: CATGACACTCTGCAACCAAGTCTTC |
|  | Reverse: GAGCAAAGGCTGCTGGTTTCA |
| *CCL4* | Forward: GAGACCAGCAGTCTTTGCTCCA |
|  | Reverse: GGAGCTGCTCAGTTCAACTCCA |
| *CCL5* | Forward: GGAGTATTTCTACACCAGCAGCAAG |
|  | Reverse: GGCTAGGACTAGAGCAAGCAATGAC |
| *β-Actin* | Forward: CATCCGTAAAGACCTCTATGCCAAC |
|  | Reverse: ATGGAGCCACCGATCCACA |

**Supplementary Figure 1.** Gating strategy used for T cells. Murine pulmonary T cells are identified by gating on the lymphocyte population and excluding doublets using forward scatter, dead cells are excluded using LIVE/DEAD Fixable Near-IR Dead Cell Stain Kit, CD3^+^ T lymphocytes are gated and further selected as CD4^+^ and CD8^+^ T cells are then gated as shown.

**Supplementary Figure 2.** Curves for the OTUs obtained from 4 groups. Species accumulation curves. The rarefaction curve reflects the rationality of the amount of sequencing data and the abundance of species in the four groups.

**
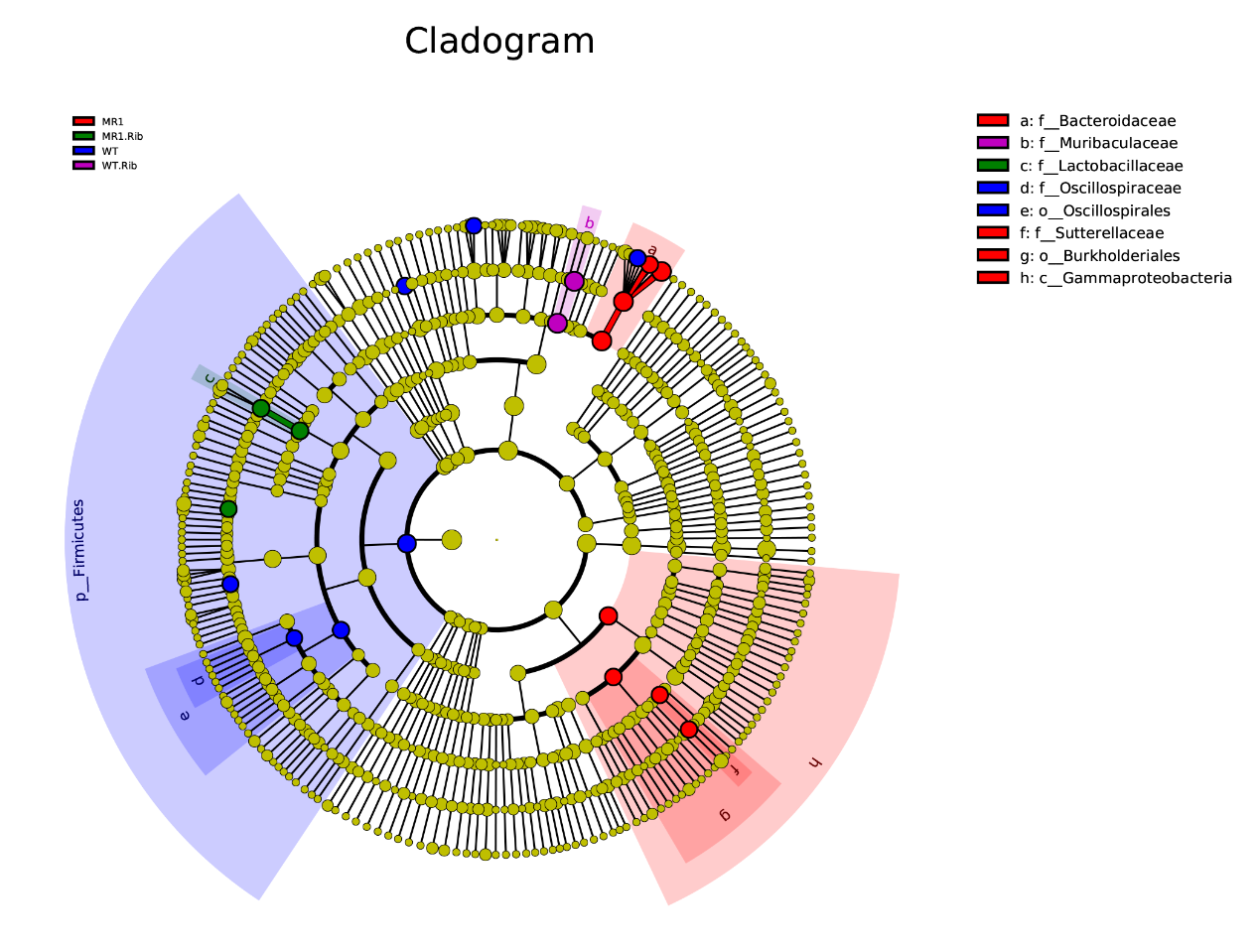
**

**Supplementary Figure 3.** Cladogram of the LEfSe analysis of the gut microbiota in different groups. Cladogram radiating from the outside to the inside each circle represents the taxonomic level from species to phylum and the classification under each level.

**
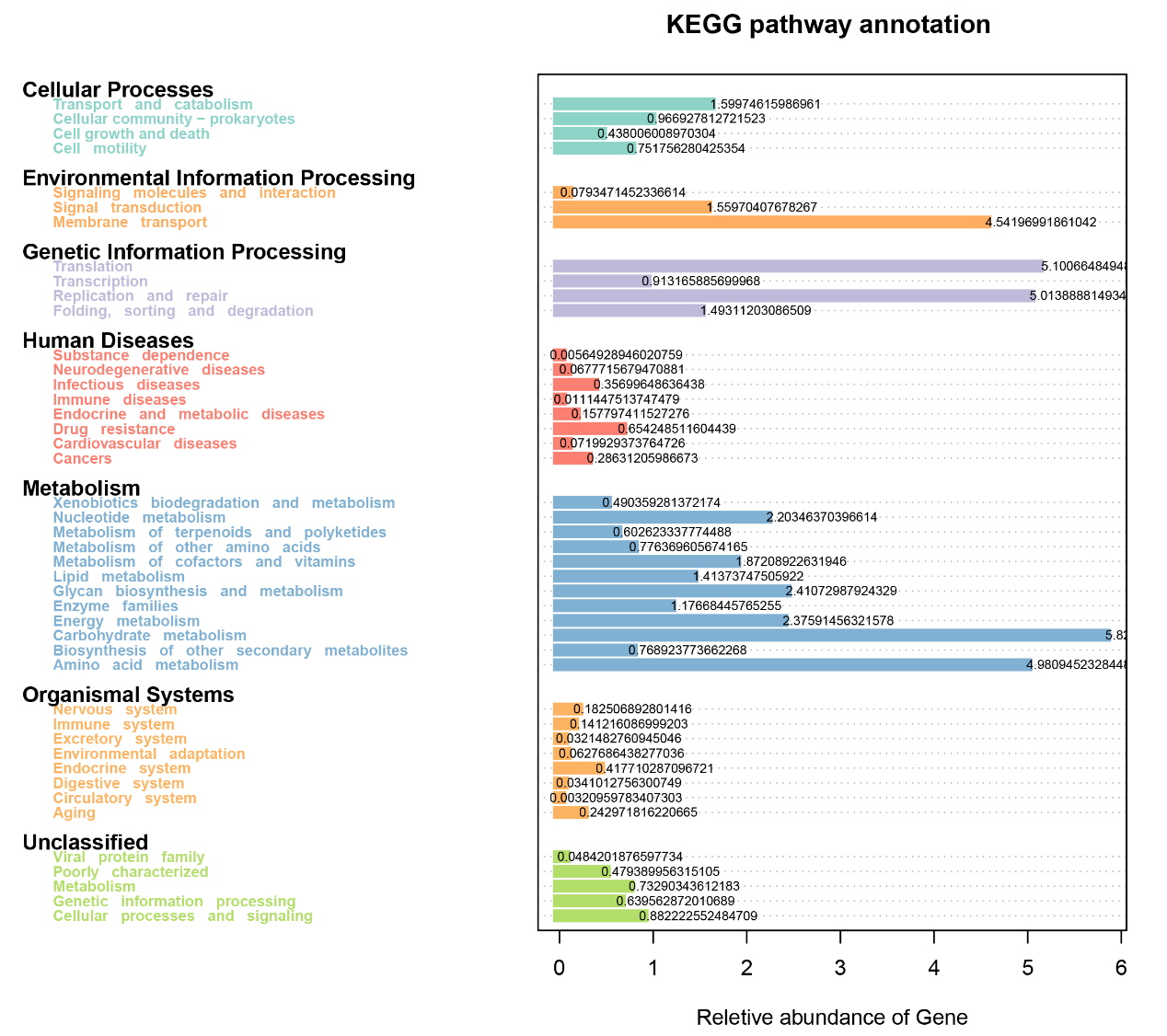
**

**Supplementary Figure 4. Classification of KEGG metabolic pathways.** The numbers on the bar chart represent the annotated genes number. The remaining axis is the code for each level function class in the database.


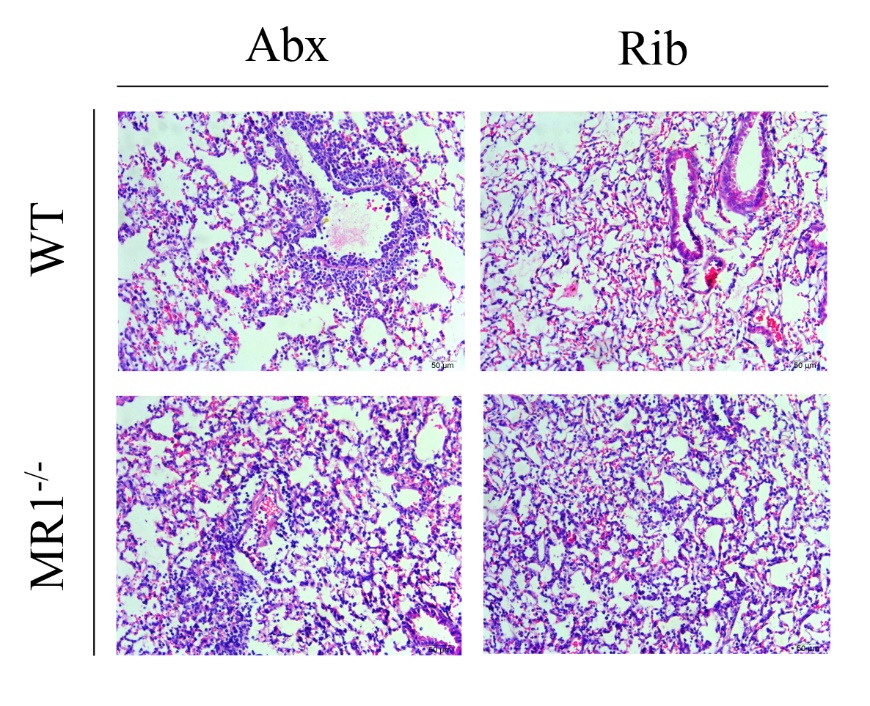


**Supplementary Figure 5.** Histologic hematoxylin and eosin stained sections from riboflavin-treated mice and WT mice. One representative image from each group was shown (scale bars, 50 µm).
